# The Presence of Background Noise Extends the Competitor Space in Native and Non‐Native Spoken‐Word Recognition: Insights from Computational Modeling

**DOI:** 10.1111/cogs.13110

**Published:** 2022-02-21

**Authors:** Themis Karaminis, Florian Hintz, Odette Scharenborg

**Affiliations:** ^1^ Department of Psychology Edge Hill University; ^2^ Department of Psychology of Language Max Planck Institute for Psycholinguistics; ^3^ Multimedia Computing Group Delft University of Technology

**Keywords:** Spoken‐word recognition, Phonological competition, Competitor space, Noise, Non‐native listening, Computational modeling, Neurocomputational model, Deep neural networks

## Abstract

Oral communication often takes place in noisy environments, which challenge spoken‐word recognition. Previous research has suggested that the presence of background noise extends the number of candidate words competing with the target word for recognition and that this extension affects the time course and accuracy of spoken‐word recognition. In this study, we further investigated the temporal dynamics of competition processes in the presence of background noise, and how these vary in listeners with different language proficiency (i.e., native and non‐native) using computational modeling. We developed ListenIN (Listen‐In‐Noise), a neural‐network model based on an autoencoder architecture, which learns to map phonological forms onto meanings in two languages and simulates native and non‐native spoken‐word comprehension. We also examined the model's activation states during online spoken‐word recognition. These analyses demonstrated that the presence of background noise increases the number of competitor words, which are engaged in phonological competition and that this happens in similar ways intra and interlinguistically and in native and non‐native listening. Taken together, our results support accounts positing a “many‐additional‐competitors scenario” for the effects of noise on spoken‐word recognition.

## 1 Introduction

### 1.1 Phonological competition

All theories of spoken‐word recognition (e.g., Allopenna, Magnuson, & Tanenhaus, 1998; Gow & Gordon, 1995; Luce & Pisoni, 1998; Slowiaczek, Nusbaum, & Pisoni, 1987; Zwitserlood, 1989) assume that when recognizing spoken words (e.g., *shape*), phonological representations of similarly sounding words (e.g., *shade* and *cape*) compete with the target for recognition (for a review, see McQueen, 2005). This process is referred to as multiple activation or phonological competition and is affected by several factors, including, among others, the lexical frequency of the targets and competitors (Dahan, Magnuson, & Tanenhaus, 2001) and phonological neighborhood density (Chen & Mirman, 2012; Vitevitch & Luce, 1999). Furthermore, phonological competition is affected by “nativeness,” as non‐native listeners, and native listeners listening to foreign‐accented speech, show enhanced phonological competition (Broersma, 2012; Porretta & Kyröläinen, 2019; Scharenborg & van Os, 2019; Scharenborg, Coumans, & van Hout, 2018; Spivey & Marian, 1999; Weber & Cutler, 2004). Similarly, previous research has shown that the presence of background noise leads to enhanced phonological competition and a prolongation of the competition phase (Ben‐David et al., 2011; Brouwer & Bradlow, 2016; Hintz & Scharenborg, 2016).

Scharenborg et al. (2018; see also Scharenborg & van Os, 2019) suggested that the effects of background noise on phonological competition arise because noise decreases the intelligibility of the speech signal (see also García Lecumberri, Cooke, & Cutler, 2010 for a review) and engages additional spurious candidate words, which would not compete under ideal listening conditions, in phonological competition. The proposal by Scharenborg et al. (2018) was based on results from an offline spoken‐word identification task, in which adult participants with native and non‐native knowledge of English listened to English words in clean listening conditions or in conditions where parts of the words were masked with speech‐shaped background noise. When listening to words in background noise, participants produced a higher number of unique misperception errors per incorrectly identified word, a measure that was taken as a proxy for the number of words involved in phonological competition. The offline spoken‐word recognition task thus provided evidence in favor of a “many‐additional‐competitors scenario” (Chan & Vitevitch, 2015). The alternative “single‐strong‐competitor scenario,” according to which the noisy speech signal partially matches with a single alternative word (Chan & Vitevitch, 2015), was not supported by the offline data of Scharenborg et al. (2018).

The number of unique misperception errors in Scharenborg et al. (2018) is by definition a proxy, which does not reveal how many or which (additional) candidates are involved in phonological competition; nor does it reveal the temporal dynamics of competition. With regard to temporal dynamics, many studies have examined online spoken‐word recognition using the “visual‐world” eye‐tracking paradigm (Huettig, Rommers, & Meyer, 2011). In this paradigm, eye movements are recorded as participants are listening to target words while looking at objects of the target and competitor words. Phonological competition is reflected in a bias in looks to objects that phonologically overlap with the target (e.g., in phonological onset or offset). Previous studies have shown that, when background noise is added to the speech signal, looking preferences for competitor words persist for a longer time window, suggesting increased phonological competition (Ben‐David et al., 2011; Brouwer & Bradlow, 2016; Hintz & Scharenborg, 2016). However, importantly, this paradigm does not shed light on what causes recognition errors to occur in spoken‐word recognition. Specifically, it is not possible to distinguish between a “many‐additional‐competitors” and a “single‐strong competitor” scenario since the visual‐world paradigm is typically constrained by a small number of objects (or words, typically four) that are presented to participants in one display (see Poretta & Kyröläinen, 2019, for a combination of offline transcription and online visual world paradigm tasks, which provided evidence that foreign‐accented speech increases the competitor space and prolongs the competition phase).

Another way to study the effects of noise on offline and online spoken‐word recognition is to use computational modeling. Computational modeling enables researchers to implement and test the viability of theoretical proposals and generate novel testable predictions based on theories. Furthermore, computational modeling approaches enable researchers to examine explicitly the internal states of the system (e.g., spoken‐word recognition) while carrying out a task, something that is more challenging empirically. In this study, we used a computational modeling approach to study the effects of background noise on phonological competition. More precisely, we sought to gather evidence that would allow us to distinguish between the “many‐additional‐competitors” and the “single‐strong competitor” scenario for the effects of noise on spoken‐word recognition (Chan & Vitevitch, 2015).

### 1.2 The present study

#### 1.2.1 Aims

The present study had two specific aims. The first aim was to examine whether the effects of noise on spoken‐word recognition, that is, the increase in misperception errors in offline spoken‐word identification and the prolongation of the competition phase in online spoken‐word recognition (e.g., Hintz & Scharenborg, 2016; Scharenborg et al., 2018), are driven by the same underlying mechanisms, in particular, whether they arise under a “many‐additional ‐competitors” or a “single‐strong‐competitor” scenario (Chan & Vitevitch, 2015). The second aim was to investigate whether the underlying mechanisms of enhanced phonological competition in the presence of background noise are similar in native and non‐native listeners. Being inclusive of individual differences related to language proficiency is important for offering an account for the effects of noise on spoken‐word recognition less skewed toward “artificial normality”, the tradition of grounding psycholinguistic theories in optimal conditions (Mattys & Liss, 2008; Mattys, Davis, Bradlow, & Scott, 2012; see also Weber & Scharenborg, 2012 for a discussion on computational models).

#### 1.2.2 Modeling approach and key assumptions

To address the two aims of this study, we developed a computational model, ListenIN (Listening‐In‐Noise), based on the neural‐network modeling framework. ListenIN models spoken‐word recognition as a task that involves mapping phonological forms onto meanings or “semantics” (similar to Gaskell & Marslen‐Wilson, 1997; McClelland & Elman, 1986; Plunkett, Sinha, Møller, & Strandsby, 1992; Shook & Marian, 2013; Smith, Monaghan, & Huettig, 2013, 2017; Zhao & Li, 2010). Phonological forms are provided as input activation to the ListenIN neural network, and ListenIN should produce appropriate semantics as output.

Knowledge of phonology‐to‐semantics mappings is acquired in two languages and through an implemented learning process. This is similar to bilingual models like BLINCS (Shook & Marian, 2013) and DEVLEX‐II (Zhao & Li, 2010) (see also speech‐perception/spoken‐word recognition models Gaskell & Marslen‐Wilson, 1997; Smith et al., 2013, 2017), rather than the connectionist models BIA+ (Dijkstra & Van Heuven, 2002) and Multilink (Dijkstra et al., 2019). The training set consists of distributed representations of phonological forms and semantics of words, which are constructed (“hardwired”) to encapsulate psychologically plausible similarities between individual words. Through training, ListenIN develops abstracted lower‐dimensionality representations, which, however, retain the similarity structure of the original representations. To this end, ListenIN employs an autoencoder neural network, in particular, a “deep” version of the architecture of Plunkett et al. (1992) model for vocabulary growth. In addition to developing abstracted representations of phonological forms and meanings, the autoencoder architecture acquires mappings of phonological forms onto meanings, without being trained explicitly (supervised training) on these mappings (unlike BLINCS, Shook & Marian, 2013, and DEVLEX‐II, Zhao & Li, 2010, which are based on self‐organizing maps and include supervised training for mappings between different types of linguistic representation).

Fully trained networks are tested on spoken‐word recognition. Spoken‐word recognition in the presence of background noise is studied by examining how well these networks map slightly distorted phonological forms onto their meanings.

With regard to native and non‐native spoken‐word recognition, a key assumption of ListenIN is that native and non‐native spoken‐word recognition systems rely on identical processing architectures, which are differentiated only through training (Filippi, Karaminis, & Thomas, 2014; Gong, Cooke, & García Lecumberri, 2015; Scharenborg & van Os, 2019). We, therefore, created two versions of ListenIN that emulated human native or non‐native lexical competence, respectively, and used these to investigate the effects of noise in experimental data from native and non‐native listeners. The only difference between the native and non‐native versions of ListenIN was their exposure to different training materials (“linguistic environments”).

#### 1.2.3 Study design

We first used ListenIN (model presented in Section 2) to simulate the offline spoken‐word identification task of Scharenborg et al. (2018) and simulate the effects of background noise on the performance of native and non‐native listeners (simulation A, presented in Section 3). Next (simulation B, presented in Section 4), we used ListenIN to simulate an online visual‐world paradigm to reproduce the effects of noise on the looking behavior of native listeners in Hintz and Scharenborg (2016) and non‐native listeners in new data we collected for the purposes of this study.

Having established that ListenIN captures offline and online spoken‐word recognition performance, we analyzed the activation states developed across the model's neural network during online spoken‐word recognition (Mechanistic analysis, presented in Section 5). Here, we considered competition from all words in the model's vocabulary rather than four words as in most visual‐world paradigm experiments to examine how the presence of noise affected phonological competition (i.e., to differentiate between “many‐additional‐competitors” vs. “single‐strong‐competitor” accounts).

## 2 The model

### 2.1 Architecture

**Fig 1:**
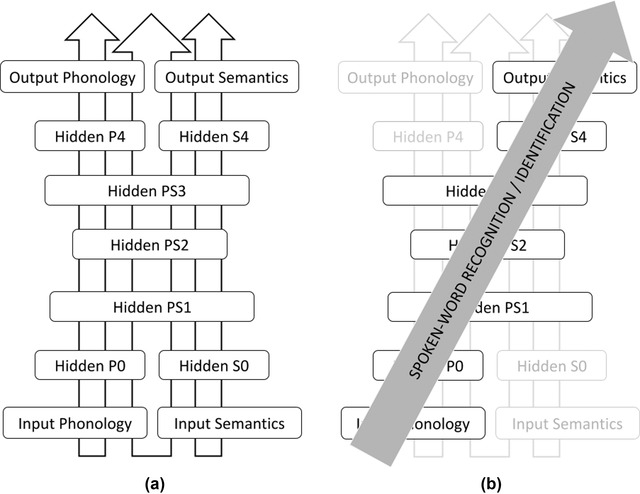
ListenIN's architecture and modeling of spoken‐word recognition. (a) During training, the autoencoder architecture is trained on representations of phonological forms and/or semantics. (b) ListenIN is tested on spoken‐word recognition. Testing focuses on the spread of activation in the parts of the network shown with black color (and ignores gray parts).

ListenIN is based on the deep autoencoder architecture shown in Fig. 1a. This architecture comprises a phonological and a semantics pathway and is trained in a semi‐supervised way on multidimensional representations of phonological forms and semantics. During training (Fig. 1a), the network is presented with three types of input‐output mapping: between phonological forms; between semantics; and between phonological forms and their semantics presented simultaneously. Training enables the autoencoder to develop abstracted representations of the input phonological forms and/or semantics representations. The abstracted representations are encoded in latent patterns in the weighted connections of the neural network and are of lower dimensionality than the original representations (e.g., “bottleneck” layer PS2). Through training, the autoencoder neural network also develops the ability to map phonological forms onto their meanings, despite the fact that it never receives explicit training on these mappings. These novel mappings are used to assess spoken‐word recognition (Fig. 1b). In particular, the autoencoder is presented with phonological forms only (no semantic input) and is tested on its ability to generate appropriate semantics in the output layer.

The parameters of the autoencoder network in the current version of ListenIN are described in Supplementary Material S1.

### 2.2 Training set and representations

The training set of the model consisted of 121 English words, taken from Scharenborg et al. (2018) and their 121 Dutch translations. In its current version, ListenIN only processes monosyllabic and disyllabic words (and as a result five word pairs used in the original study were excluded from ListenINs training set). English and Dutch translation equivalents had different phonological forms and were assumed to have exactly the same semantics (but see Pavlenko, 2009; Zhao & Li, 2010 for accounts positing that word semantics differ across languages and Dong, Gui, & MabWhinney, 2005 for accounts suggesting dynamic and asymmetric bilingual representations).

We developed feature‐based schemes for the representation of phonological forms and their semantics, which encapsulated psycholinguistically plausible phonological and semantic similarities between individual words. The phonological and semantic representations are explained in what follows.

#### 2.2.1 Phonological representations

The phonological forms of the words were represented using a binary feature‐ and slot‐based representational scheme, presented in detail in Supplementary Material S2.

Phonological forms were presented as phoneme sequences, with individual phonemes fitted within a 13‐slot disyllabic word template: CCCVVCCCVVCCC (C: consonant; V: vowel). We placed phonemes in this template using an alignment‐to‐the‐left strategy for syllables (similar to Shook & Marian, 2013; Smith et al., 2013, 2017). For example, *carrot* (SAMPA: kh.{.r.@.t.) was aligned as follows: kh _ _ { _ r _ _ @ _ t _ _). The phonological representations also included prosodic information, namely, syllabic length and syllabic stress. Syllabic length was represented using thermometer encoding over 2 bits (monosyllabic = 01; disyllabic = 11), while syllabic stress was represented with localist (one‐hot) encoding over two bits. Finally, language‐membership information (two localist units, English or Dutch) was also included in phonological representations. In sum, the phonological forms of words were represented using a 292‐bit vector with an average of 25.94 ± 6.31 “ones” per word (a sparse vector).

The use of a disyllabic template with alignment to the left enabled the model to accommodate aspects of the incremental nature of speech processing by encoding “time into space.” This way, it was possible to capture phonological similarities between individual words within a syllable irrespective of the ordering of phonemes (e.g., similarities between *tap* and *trap*, example from Shook & Marian, 2013, p. 307). However, some other similarities (e.g., rhymes *tap* and *recap*) were missed out with this scheme, which could not capture phonological similarities between syllables of a different order (the first syllable of *tap* with the second syllable of *recap*).

The alignment‐to‐the‐left strategy also enabled us to set up ListenIN so that word onset information would be more important for word‐offset information in spoken‐word recognition (Allopenna et al., 1998; Marslen‐Wilson & Zwitserlood, 1989; McQueen & Huettig, 2012). More specifically, the alignment‐to‐the‐left strategy implied that all words (monosyllabic and disyllabic) occupied slots at the leftmost part of the phonological template, but only disyllabic words (45 Dutch and 39 English) occupied slots at the rightmost part of the template. As a result, the information corresponding to the leftmost slots of the template was always available and always used to distinguish between a given input phonological representation and input phonological representations of all other words. By contrast, information corresponding to the rightmost part of the template was provided optionally (in disyllabic words only) and was used to distinguish between the phonological representations of a subset of the words which occupied these slots. This asymmetry implied that information in the leftmost part of the template, associated with the onset of words, was especially important and more influential on word recognition compared to information in the rightmost part of the template (associated with the offset of bisyllabic words).

**Fig 2:**
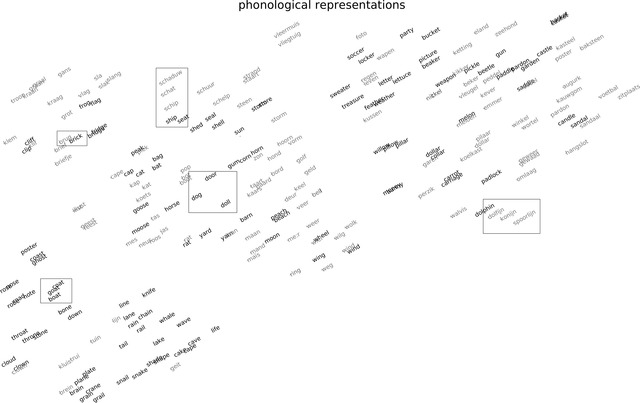
Visualization of words in the training set of ListenIN based on the similarity of their phonological representations. Visualization was made using the t‐SNE technique (t‐Distributed Stochastic Neighbour Embedding; van der Maaten & Hinton, 2012). Black ink shows English words; gray ink shows Dutch words. Rectangles indicate examples of clusters of phonologically related words.

To gain some insight into the phonological regularities and the similarity structure incorporated in the phonological representations used in ListenIN, we created a two‐dimensional visualization of the phonological vectors for English (black) and Dutch (gray) words, which is shown in Fig. 2. This visualization was produced using the t‐Distributed Stochastic Neighbor Embedding technique (t‐SNE; van der Maaten & Hinton, 2012), a method that optimizes the visual presentation of between‐vectors similarity structure. There are several clusters of phonologically similar words within each language, for example, words with similar onsets (e.g., English: *dog*, *door*, *doll*; Dutch: *schaduw* (shadow), *schat* (treasure), *schip* (ship), with the English translation equivalent *ship* in proximity), as well as rhyming words (e.g., English: *boat, coat, goat*; Dutch: *spoorlijn* (rail), *dolfijn* (dolphin), *konijn* (rabbit)). There is also a remarkable overlap between English and Dutch phonological forms, though English and Dutch phonological representations that are close to each other are not necessarily translation equivalents (e.g., *tail* and *tuin* (garden)).

#### 2.2.2 Semantics representations

The meanings of words in ListenIN were represented using distributed representations, that is, vectors that encoded semantic similarities between individual words. We used a 300‐dimensional binary scheme, which represented the meanings of words with an average of 52.73 ± 11.48 ones “over” a 300‐bit vector.

The distributed semantics representations of ListenIN were derived from an existing 300‐dimensional model of semantic similarity, in particular, a word‐embedding model trained on a corpus of 100 billion words from Google News (word2vec, “GoogleNews‐vectors‐negative300.bin”; Mikolov, Sutskever, Chen, Corrado, & Dean, 2012; model retrieved from the gensim Python library, Řehůřek & Sojka, 2010). Word‐embedding models are distributional‐semantics models, which are built from large text corpora based on psychologically plausible learning principles and can account for human performance in psycholinguistic tasks (Mandera, Keuleers, & Brysbaert, 2017). The original word‐embedding model (“GoogleNews‐vectors‐negative300.bin”; Mikolov et al., 2012) was real‐numbered, and pilot simulations suggested that the ListenIN autoencoder network could not learn to map phonological (which were binary vectors) onto real‐numbered semantic representations. We, therefore, transformed the real‐numbered word‐embedding representations in the initial word2vec model to binary vectors by setting all values lower than −0.175 to 1, and all other values to zero. The threshold of −0.175 was chosen arbitrarily to yield binary semantics vectors that were sparse (similarly to the phonological representations), while maintaining a considerable portion of the meaningful structure in the real‐numbered representations.

**Fig 3:**
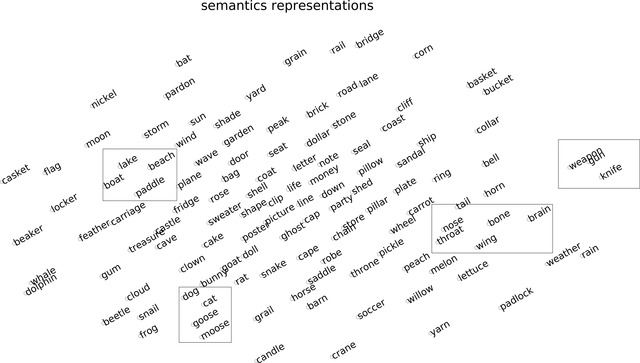
Visualization of the semantic representations for the words in the training set of ListenIN, using the t‐SNE method (van der Maaten & Hinton, 2012). Rectangles indicate examples of clusters of semantically related words.

For the resulting binary distributed representations, the semantic similarities between individual words are illustrated in Fig. 3, which again used the t‐SNE method (van der Maaten & Hinton, 2012) for two‐dimensional visualization. There are several clusters of related meanings in this plot (e.g., *cat, goose, moose, bunny; boat, lake, paddle, beach; nose, throat, tail, bone, wing, brain;* or *weapon/gun/knif*e).

### 2.3 Training the neural network

The autoencoder was trained by integrating two training techniques for deep neural networks, namely, weight initialization with pretraining (Hinton & Salakhutdinov, 2006) and weight fine‐tuning with injection of noise (Vincent, Larochelle, Bengio, & Manzagol, 2008; Zur, Jiang, Pesce, & Drukker, 2009), with a training regime that considered three phases of learning based on Plunkett et al. (1992). We blended these techniques based on pilot experiments, which showed that these techniques allowed the neural network to learn to map phonological forms onto semantics and show abilities for word comprehension (mapping an input phonological form to appropriate semantics) without receiving explicit training on these mappings. The training of the autoencoder is detailed in Supplementary Material S3.

### 2.4 Experimental setup for simulations A and B

To simulate spoken‐word identification (simulation A) and the visual‐world eye‐tracking experiments (simulation B), we used a two‐step experimental setup, consisting of an *Exposure* and a *Test* step.

#### 2.4.1 Exposure step

In the Exposure step, we developed native and non‐native versions of ListenIN with knowledge of English and Dutch, respectively, through asymmetric exposure to English and Dutch words during the training phase (weight fine‐tuning with interleaving three training phases). In particular, we developed 50 training replications (with different pseudorandom seeds for weight initialization) of two versions of the model:

- networks with knowledge of a bilingual vocabulary of English and Dutch translation equivalents, however, with less robust knowledge of Dutch compared to English words (“native English/non‐native Dutch” listener, exposure ratio English : Dutch = 3 : 1);
- networks with knowledge of a bilingual vocabulary of English and Dutch translation equivalents, however, with less robust knowledge of Dutch compared to English words (“native Dutch/non‐native English” listener; exposure ratio English : Dutch = 1 : 3).

We note that the two versions of ListenIN were exposed to the same number of phonological form‐meaning mappings, implying that any performance differences would arise from the differential exposure to the two vocabularies. However, we have replicated all our results with an alternative implementation of ListenIN, in which native listeners are modeled with the neural network being exposed to a monolingual vocabulary (see Supplementary Materials S4 and S5).

#### 2.4.2 Test step

In the Test step, trained networks from the two versions of the model were tested on simulated spoken‐word identification and eye‐tracking tasks, which were parallel to the human spoken‐word recognition experiments. Depending on the target language of the spoken‐word experiment (which was either English or Dutch) the “native English/non‐native Dutch” version and the “native Dutch/non‐native English” were used to model the corresponding native or non‐native human data.

The Test step yielded model‐based measures of spoken‐word recognition performance. These measures were derived from the activation patterns in the output semantics layer of ListenIN following the presentation of a phonological form in the input layer based on task‐specific procedures, which are detailed in the sections presenting simulations A and B. The model‐based measures were comparable with corresponding measures from the human data in the offline spoken‐word recognition experiment (Scharenborg et al., 2018) and the visual world‐paradigm (Hintz & Scharenborg, 2016).

### 2.5 Comparisons between model‐based and human measures

We compared data referring to model‐based measures from ListenIN and human measures in four ways:

#### 2.5.1 Parallel statistical analyses

First, we examined if model‐based and human measures presented the same empirical effects on a given measure, for example, if they presented an effect of noise or nativeness. To this end, we analyzed the model‐based and human data, using the same task‐specific statistical procedures.

#### 2.5.2 Pearson correlation coefficients for the overall fit to data

Second, we assessed the overall fit of the ListenIN output to the human data on a given measure. To this end, we used Pearson correlation coefficients. The correlation coefficients were calculated using averaged values across trained network replications and noise repetitions for the model, and across participants for the human data. In the comparisons on a given measure, we used two vectors (one for the model and one for the human data) with aggregated values for all individual levels of all different factors (e.g., noise intensity and nativeness) considered in a given task.

#### 2.5.3 Pearson correlation coefficients focusing on individual factors

Third, we assessed the fit of ListenIN to the human data focusing on changes in model‐based and human measures related to individual factors (rather than the overall fit to the aggregated data, as in 2.5.2). These comparisons enabled us to evaluate ListenIN's ability to capture the effect of individual factors (e.g., noise intensity) on a given measure.

The focused comparisons were based on correlation coefficients, which now compared the differences in model‐based and human measures between the levels of a factor in focus (with one level treated as the baseline). For example, to examine the fit to the human accuracy data focusing on the effect of nativeness, we compared two vectors (one for the model and one for the human data) aggregating differences in accuracy between native and the non‐native listening conditions for all individual levels of all other factors (e.g., all individual noise intensities), which were were kept unchanged in this analysis. The quantitative comparisons thus yielded fit‐to‐data measures focusing on the effects of individual factors.

#### 2.5.4 Baseline measures from an Input‐based model

Finally, the fit of model‐based measures from ListenIN to human measures was examined with reference to the fit of a so‐called Input‐based model, which was driven only by the characteristics of the input phonological representations and involved no training. In this model, the response to an input phonological pattern was simply the vocabulary word with the closest matching phonology (and not an output semantics pattern produced after passing input activation through a neural network). The rationale for using the Input‐based model was to examine the extent to which the distributed input phonological representations, which (as discussed in Section 2.2.1) were constructed (“hardwired”) to encapsulate phonological similarities between individual words, could account for human data in simulations A and B. This was in isolation from any abstracted linguistic knowledge such as that acquired by ListenIN through learning and encoded in the weighted connections of the neural network. In effect, the Input‐based model enabled us to assess how much the performance of ListenIN was driven from the input phonological representations and whether a learning process leading to abstracted linguistic knowledge was necessary to simulate the human data.

The fit of measures from the Input‐based model to the human data was assessed with Pearson correlation coefficients for the overall fit and for the effects of individual factors (i.e., similar to ListenIN).

## 3 Simulation A: Offline spoken‐word identification

Karaminis & Scharenborg (2018) showed that ListenIN can simulate offline spoken‐word identification performance of native and non‐native listeners. This section extends this earlier computational modeling work to include simulations with the Input‐based model, as well as more simulation replications and more detailed comparisons with the human data.

### 3.1 Summary of target empirical data from Scharenborg et al. (2018)

**Fig 4:**
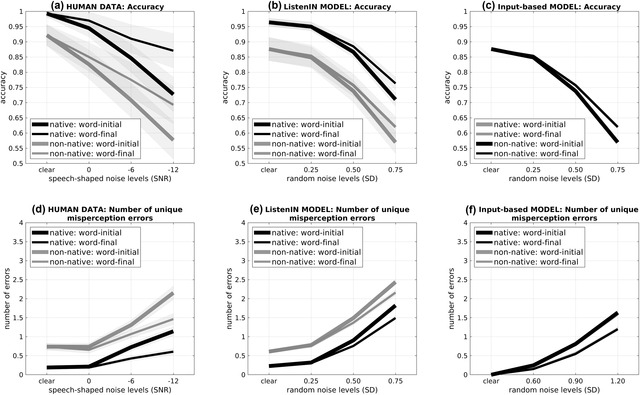
Target empirical data (panels a and d) and modeling results from ListenIN and the Input‐based model (panels b, c, e, and f) in offline spoken‐word identification. The top panels (a–c) present overall accuracy for different noise intensities; the bottom panels (d–f) present the number of unique misperception errors for different noise intensities. Noise intensity refers to SNR values for speech‐shaped noise in the human data; and to the standard deviation of the added noise in ListenIN and the Input‐based model. Black ink shows the performance of native listeners; gray ink shows the performance of non‐native listeners (black and gray lines overlap in c and e). Thick lines correspond to the word‐initial noise condition; thin lines correspond to the word‐final noise condition. Error bars show 1 SEM.

The target data from Scharenborg et al. (2018) included two dependent variables: (1) overall word‐identification accuracy and (2) the number of unique misperception errors per incorrectly identified word (a proxy for the size of the competitor space). These two measures were examined in four different listening conditions (in clean and in three signal‐to‐noise ratios [SNRs] of stationary speech‐shaped noise: 0, −6, and −12 dB), in two different conditions for the position of noise (word‐initial and word‐final masking), and in two listener groups (native and non‐native). Fig. 4 shows the human data on accuracy (panel a) and the number of unique erroneous responses (panel d) from Scharenborg et al. (2018).

With regard to accuracy, Scharenborg et al. (2018) reported significant main effects of noise intensity, noise position, and listener group: Accuracy was lower in higher noise intensities, in the word‐initial than the word‐final masking condition, and in non‐native compared to native listening.

Turning to the number of unique misperception errors (proxy of how many words were involved in lexical competition), Scharenborg et al. (2018) reported main effects of intensity, noise position, and group. The number of unique misperception errors was higher in higher noise intensities, higher in the word‐initial than the word‐final condition, and higher in non‐native compared to native listeners. Accuracy rates and the number of unique misperception errors were affected in inverse ways by the conditions of the spoken‐word identification task (the number of unique errors increased when accuracy dropped).

### 3.2 Simulation design

#### 3.2.1 Exposure step

In the Exposure step, we developed “native English/non‐native Dutch” versions (model trained on English and Dutch translation equivalents with a 3 : 1 ratio) and “non‐native English/native Dutch” versions (model trained on English and Dutch with a 1 : 3 ratio) of ListenIN. The former were used to model data from native listeners and the latter were used to model non‐native listening conditions.

#### 3.2.2 Test step

In the Test step, we tested the native and non‐native English versions of ListenIN in a simulated spoken‐word identification task, similar to the original study. To simulate the offline spoken‐word identification results of Scharenborg et al. (2018), we presented the fully trained networks with phonological representations of words (no semantics), which included only the 13‐slot disyllabic word template and the Dutch language unit (corresponding to the assumption that the language of the task is known). Prosodic information (syllabic length and stress) was not used as input to ensure that these cues (for which we avoided making assumptions on how they are affected by the presence of background noise) did not interfere with the model's performance in the simulated offline spoken‐word recognition task.

In this simulation, ListenIN's response was evaluated by examining whether the model produced the appropriate semantics in the output layer (Fig. 1b). The output semantics were evaluated with a nearest neighbor criterion: they needed to be closer in the Euclidean space to the semantics of the presented word than any other semantics pattern. If this was not the case, the closest match was taken to be the word that ListenIN (mis)recognized and this response was recorded.

To be parallel to the conditions of the human‐listener experiment, we included a zero‐noise condition, three levels of noise with different intensity, and conditions of word‐initial versus word‐final noise. Noise was implemented as a real‐numbered vector selected from a Gaussian distribution with mean = 0 (clear) and *SD* = 0.30 (low intensity), 0.60 (medium intensity), or 0.90 (high intensity), which was added only to slots occupied by a given word. For all stimulus words, word‐initial and word‐final noise was applied to the same phones as in Scharenborg et al. (2018). The value for the *SD* of the random noise was a free parameter of the model. Its value was set so that ListenIN matched human spoken‐word identification accuracy in the word‐initial noise condition.

### 3.3 Results

The middle and right panels of Fig. 4 present the modeling results from ListenIN and the Input‐based model on accuracy rates (panels b and c, correspondingly) and the number of different erroneous responses provided for a given word stimulus (panels e and f).

#### 3.3.1 Accuracy

An analysis of ListenIN's accuracy with generalized linear mixed‐effect models (Baaynen, Davidson, & Bates, 2008) showed main effects of noise intensity (*β* = 1.29, *SE* = 0.09, *p* < .001), position (*β* = −0.47, *SE* = 0.08, *p* < .001), and group (*β* = −0.39, *SE* = 0.05, *p* < .001). ListenIN thus generated all the main effects for the human accuracy data described by Scharenborg et al. (2018) as accuracy was lower in higher levels of noise, in the word‐initial compared to the word‐final noise condition, and in the non‐native compared to the native version.

In terms of quantitative comparisons between the modeling results (panel b) with the human data (panel a), the Pearson correlation coefficient was *r*(16) = .96, *p* < .001, suggesting an excellent fit. Pearson correlation coefficients comparing differences between the levels of noise intensity also suggested an excellent fit to the human data, *r*(12) = .91, *p* < .001. The same holds for correlation coefficients comparing differences between the levels of noise position, *r*(8) = .98, *p* < .001, and group, *r*(8) = .93, *p* < .001, on accuracy.

The Input‐based baseline model (panel c) was also successful in fitting the human accuracy data, *r*(16) = .81, *p* < .001, and captured the differences between the levels of noise intensity and position (*p*s < = .001). However, the Input‐based baseline model did not capture differences between the two levels of group (native vs. non‐native) with regard to accuracy, *r*(8) = −.35, *p* = .39. This was an expected result as the baseline model did not include a learning process that would differentiate between native and non‐native performance but instead relied on the input phonological representations.

#### 3.3.2 Misperception errors

An analysis of misperception errors with generalized linear mixed‐effect models (Baaynen et al., 2008) showed main effects of noise intensity (*β* = −0.92, *SE* = 0.02, *p* < .001), position (*β* = 0.25, *SE* = 0.04, *p* < .001), and group (*β* = 0.08, *SE* = 0.04, *p* = .015) on misperception errors. That is, ListenIN reproduced the key effects from the human data: larger numbers of unique erroneous responses (1) as noise increased, (2) for word‐initial compared to the word‐final noise condition, and (3) in the non‐native compared to the native version.

The Pearson correlation coefficient between human data and modeling results on the number of unique misperception errors was *r*(16) = .92, *p* < .001, suggesting an excellent fit. The model also captured differences between the levels of noise intensity, *r*(12) = .93, *p* < .001, noise position, *r*(8) = .96, *p* < .001, and group, *r*(8) = .72, *p* = .043.

The Input‐based baseline model also fitted the human misperception errors data, *r*(16) = .74, *p* < .001, capturing differences between the levels of noise intensity, *r*(12) = .93, *p* < .001, and position, *r*(8) = .96, *p* < .001; however, it did not capture the effects of group, *r*(8) = −.34, *p* = .41.

### 3.4 Conclusion

Simulation A showed that ListenIN can account for human performance in an offline spoken‐word identification task (Scharenborg et al., 2018) capturing the effects of noise on word identification accuracy and on the number of unique misperception errors and changes in these two dependent variables in word‐initial versus word‐final noise conditions, and in native versus non‐native listening groups.

## 4 Simulation B: Online spoken‐word recognition

Having replicated the offline results of Scharenborg et al. (2018), in simulation B, we set out to establish whether ListenIN simulates the effects of noise on phonological competition in online task settings. To that end, we used ListenIN to simulate the visual‐world eye‐tracking results reported by Hintz and Scharenborg (2016), who tested Dutch native speakers on a speech‐in‐noise task in their mother tongue. To be parallel to the offline study by Scharenborg et al. (2018) and simulation A, which investigated the effects of noise in both native and non‐native listeners, we complemented the data of Hintz and Scharenborg (2016) with new data from non‐native Dutch listeners (German speakers). We used the same stimuli and the same parameter settings as in simulation A and designed a simulated version of the visual‐world paradigm to evaluate ListenIN's ability to account for human looking preferences in the data of Hintz and Scharenborg (2016) and the new data.

### 4.1 Online task and target empirical phenomena

#### 4.1.1 Visual‐world paradigm

In the online spoken‐word recognition experiment, participants listened to Dutch target words embedded in semantically neutral, and nonpredictable carrier sentences. For example, they listened to the Dutch word *zwan* in “Hij dacht direct aan een *zwaan* toen Bob over ganzen begon te praten” (“*He immediately thought of a swan when Bob started talking about geese*”). The sentences, including target words, were presented in clean listening conditions and masked with stationary speech‐shaped noise at two SNRs: +3 and −3 dB. During the auditory presentation of sentences, participants looked at a display which showed four pictures. Participants had 3 s to inspect these pictures before listening to the carrier sentences.

There were two main conditions for the objects depicted in the display, the *target condition* and the *phonological onset competitor condition*. In the target condition, the display included an object depicting the target word, for example, *zwaan* (*swan*) and three additional objects depicting phonologically and semantically unrelated words (henceforth distractors; e.g., *toilet* (toilet); *komkommer* (cucumber); *fles* (bottle)). A bias in looks to the target (over the unrelated distractors) is taken to reflect recognition of the target word. In the phonological onset competitor condition, the display showed an object depicting a word overlapping with the target in the first few phonemes, for example, *zwaard* (sword) and three objects depicting the same three phonologically and semantically unrelated distractors as in the target‐present condition. A bias in looks to the phonological competitor is taken to reflect phonological competition between the unfolding target and similarly sounding words.

Participants’ eye movements were analyzed starting at the onset of the target words. Target words were on average 350 ms long. Looking preferences were computed by dividing looks to the critical objects by average looks to the three distractors and correcting for initial biases by subtracting the value of this ratio during a baseline time window (0–200 ms; cf. Huettig & Altmann, 2007). As it takes approximately 200 ms to program and launch a saccadic eye movement (Saslow, 1967), fixations starting 200 ms after target word onset are likely to reflect linguistic processing of the target word.

We analyzed log‐transformed looking preferences, comparing averages in the first 200 ms after target word onset with looking preferences during the time interval 200–800 ms post‐onset, divided into three equal‐sized bins. We used the same type of analysis as Hintz and Scharenborg (2016) to enable quantitative comparisons of the model to the human data (using the same number of bins). This analysis was item‐based, averaging responses for individual items across participants. Log‐transformed looking preferences for target and onset competitor objects were subjected to planned *t*‐test comparisons (one‐tailed).

#### 4.1.2 Data from native listeners from Hintz and Scharenborg (2016)

**Fig 5:**
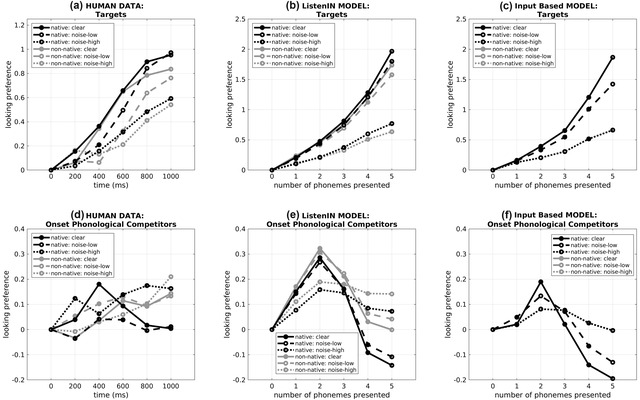
Target empirical data (panels a and e) and modeling results from ListenIN (panels b and d) and the Input‐based model (panels c and f) in online spoken‐word identification. The top panels (a–c) present looking preferences for target words; the bottom panels (d and f) present looking preferences for onset phonological competitors. Time is measured in ms in the human data and in the number of phonemes that have been incrementally presented in the modeling results. Black ink shows the performance of native listeners; gray ink shows the performance of non‐native listeners (black and gray lines overlap in panels c and f). Continuous lines correspond to the clean listening condition; dashed and dotted lines correspond to the noisy listening condition (SNR of +3 and −3 dB in humans, added random noise with a standard deviation *SD* = 0.25 and *SD* = 0.75 in ListenIN, and *SD* = 0.60 and 1.20 for the Input‐based model––settings from simulation A).

The left panels in Fig. 5 show (in black ink) the looking preferences of native human listeners from Hintz and Scharenborg (2016) for targets (panel a) and phonological onset competitors (panel d), averaged across items and participants. Data are shown for the clean (continuous lines), +3 dB SNR (dashed lines), and the −3 dB (dotted lines) listening conditions.

With regard to looking preferences for targets, when listening in the clear, fixations on the target objects were significantly more likely than fixations on the unrelated pictures in the interval 200–400 ms post‐onset, compared to the baseline window [*t*(65) = 5.89, *p* < .001 and this bias remained significant throughout the trial (*p*s < .001). This result suggested that listeners recognized the spoken target shortly after it was presented. The same was observed when the target words were masked by background noise at an SNR of +3 dB (*p*s < .001, not shown in Fig. 5). At an SNR of −3 dB, the target bias was significant in all time windows (*p*s < .001); however, the bias appeared slightly later compared to the clear condition indicating that the presence of noise delayed target word recognition.

Turning to participants’ looking preferences for the phonological onset competitors, in the clear condition, these were significantly higher than the baseline during the window 200–400 ms after target onset [*t*(65) = 2.61, *p* = .01], but the two measures did not differ in the other two time windows [400–600 ms: *t*(65) = –0.89, *p* = .19; 600–800 ms: *t*(65) = 0.44, *p* = .33]. These results suggested competition between the unfolding spoken target and the words overlapping with the target in the time window 200–400 ms post‐onset, that is, in word‐initial phonemes. In the +3 dB SNR condition, the onset competitor bias arose in the same window [*t*(65) = 2.56, *p* = .01], but was elongated, lasting until approximately 600 ms post‐onset [400–600 ms: *t*(65) = –1.83, *p* = .04; 600–800 ms: *t*(65) = –1.18, *p* = .12]. However, only a trend toward a phonological bias was observed in the −3 dB SNRcondition [200–400 ms: *t*(65) = 1.59, *p* = .06]. Thus, the presence of noise led to weaker and elongated bias for the onset‐related objects, and most likely reflected an elongation in competition resolution.

In sum, the key findings of the human data reported in Hintz and Scharenborg (2016) were that noise (1) delayed and attenuated looking preferences for targets and (2) prolonged the time window of looking preferences for onset competitors and attenuated their magnitude.

#### 4.1.3 New data from German listeners

To evaluate modeling results in non‐native listening conditions, we collected new data from 45 non‐native German listeners of Dutch (aged between 18 and 33, mean = 23.0, *SD* = 3.0). All were students at Radboud University and were paid for their participation. All participants had normal hearing and normal or corrected‐to‐normal vision and did not report a history of neurodevelopmental conditions. Participants scored on average 71.0% (*SD* = 10.0) on a Dutch non‐native language proficiency test, where 50% reflects chance level (Lemhöfer & Broersma, 2012). Participants gave informed written consent before taking part in this study, which was approved by the ethics board of Radboud University.

Non‐native participants’ eye‐tracking data are shown in the left panels in Fig. 5 in gray ink. With regard to looking preferences for targets, in the clear, the German non‐native listeners showed higher looking preferences for targets compared to baseline in the time window 400–600 ms, *t*(65) = 5.43, *p* < .001, that is, one bin later compared to Dutch listeners (suggesting some delay in target recognition during non‐native listening). In the +3 and −3 dB SNR conditions, looking preferences of non‐native listeners for targets were further delayed and arose after 600 ms post‐onset (+3 dB SNR: *t*(65) = 4.76, *p* < .001; −3 dB SNR: *t*(65) = 2.32, *p* < .001).

With regard to looking preferences for onset phonological competitors, in the clear, there was a significant bias for onset competitors only in the time window 200–400 ms, *t*(65) = 1.96, *p* = .02. Similar to the native data, this bias was delayed in the presence of background noise. At an SNR of +3 dB, non‐native listeners showed significant looking preferences for onset competitors in time window 400–600 ms, *t*(65) = 1.78, *p* = .02, that is, one bin later compared to the clear condition. Looking preferences for onset phonological competitors were not significant at an SNR of −3 dB.

In sum, non‐natives showed weaker looking preferences for targets and onset competitors compared to Dutch listeners. Similar to native listeners, noise delayed and attenuated looking preferences to targets and prolonged and attenuated phonological competition.

### 4.2 Simulation design

#### 4.2.1 Exposure test

In the Exposure step for this simulation, we developed “native Dutch/non‐native English” versions (model trained on Dutch and English translation equivalents with a 3 : 1 ratio) and “non‐native Dutch/native English” versions (model trained on Dutch and English translation equivalents with a 1 : 3 ratio). The former were used for modeling data from native Dutch listeners from Hintz and Scharenborg (2016) and the latter for modeling the newly collected data from non‐native Dutch listeners. We note that the non‐target (non‐Dutch) language was English in ListenIN and, therefore, differed from German, the native language of the human participants. However, as participants were university students in programmes taught in English, they were also likely highly proficient in English.

#### 4.2.2 Test step

To simulate the temporal dynamics of online spoken‐word recognition, we presented fully trained versions of ListenIN with phonological forms of Dutch words (and no semantics) in an incremental fashion. The incremental presentation started from the complete absence of phonological input at timestep 0 (baseline window); presenting only the first phoneme of the word at timestep 1, presenting only the first two phonemes of the word at timestep 2, and so on until the full phonological form was presented to the input layer. The Dutch language unit was not used throughout the incremental presentation of phonological words, similar to simulation A.

To simulate the target condition and the onset phonological condition of the visual‐world paradigm, we compiled test stimuli parallel to those used in Hintz and Scharenborg (2016). Each Dutch word in the training set of ListenIN was assigned an onset phonological competitor and three unrelated distractors based on Euclidean distances between phonological and semantics representations. For example, the target word *dollar* (dollar) was assigned the onset phonological competitor *dolfijn* (dolphin) and the three unrelated distractors were *maïs* (corn), *geest* (ghost), and *mand* (basket).

The phonological forms of the Dutch words were presented incrementally in the clean and in two noise conditions, with added random noise. Random noise was set at the first two noise levels of the offline experiment, that is, with *SD* = 0.30 and *SD* = 0.60 (the SNRs used in the online task of Hintz and Scharenborg, 2016, +3 and −3 dB, were on the lower side of the SNRs used in the offline task of Scharenborg et al., 2018: 0, −6, and −12 dB SNR).

We obtained model‐based measures of “looking preferences” during the incremental presentation of all Dutch words in the test set. These were derived from comparisons between Euclidean distances between the semantics generated in the output layer and the semantics representations of the different test stimuli. We assumed that in each timestep, the model “looked” at the test stimulus the semantics of which was closer in the Euclidean space to the semantics activation generated at the output layer of ListenIN. For example, to obtain model‐based looking preferences for the target condition, during the incremental presentation of the word *dollar* (dollar), we examined whether the output semantics pattern was closer to the semantics of *dollar*, or one of the three distractors *maïs* (corn), *geest* (ghost), and *mand* (basket).

We processed model‐based looking preferences and the data of Hintz and Scharenborg (2016) based on the same procedure. We divided looks to the critical objects by looks to the three distractors at each timestep, corrected for initial biases by subtracting the baseline value at timestep 0 (no input activation), and applied a logarithmic transformation.

### 4.3 Results

The middle and right panels of Fig. 5 show modeling results from ListenIN and the Input‐based model on looking preferences for targets (panels b and c, correspondingly) and for onset phonological competitors (panels e and f) for the clean (continuous lines), the *SD* = 0.25 (dashed lines) and the *SD* = 0.75 (dotted lines) noise condition, and for the native (black ink) and non‐native (gray ink) versions of the model.

#### 4.3.1 Targets

Looking preferences to targets were significant after the presentation of the second phoneme in both the native and non‐native versions of ListenIN and in clean and noisy listening conditions (*p*s < .001). The addition of noise attenuated looking preferences for targets in both native and non‐native ListenIN, while looking preferences for targets were less pronounced in the non‐native compared to the native version.

The correlation coefficient between the human looking preferences to targets (panel a) and the modeling results (panel b) was *r*(36) = .95, *p* < .001, suggesting an excellent fit––though looking preferences to targets in ListenIN are twice as high compared to the model. Correlation coefficients focusing on differences between the levels of individual factors were significant for noise intensity, *r*(24) = .67, *p* < .01, as well as nativeness, *r*(18) = .58, *p* = .012.

The comparison of the Input‐based baseline model to the human data on looking preferences for targets showed an excellent quantitative fit for the overall accuracy data, *r*(36) = .93, *p* < .001, and a good fit to the data when focusing on differences between the levels of noise intensity, *r*(24) = .58, *p* = .003. However, the baseline model could not capture differences between the two levels of nativeness on target looking preferences, *r*(18) = −.22, *p* = .39. Thus, as in simulation A, the exposure of the two versions of ListenIN to different proportions of Dutch and English words during training enabled the model to account for the effects of noise in native and non‐native listening.

#### 4.3.2 Onset competitors

With regard to simulated looking preferences for phonological onset competitors, the native version showed significant looking preferences after the presentation of the first, *t*(120) = 5.41, *p* < .001, second, *t*(120) = 5.92, *p* < .001, third phoneme, *t*(120) = 3.4, *p* < .001 in the clean listening condition. However, when random noise of an *SD* = 0.25 or *SD* 0.75 was added, significant looking preferences were prolonged and also included the fifth phoneme.

A similar delay in the looking preferences for onset phonological competitors in noise was observed in the non‐native version of ListenIN. In the clear, statistically significant looking preferences arose after the presentation of the first, second, and third phonemes (*p*s < .001). In the presence of noise, the window in which statistically significant looking preferences for onset phonological competitors occurred included phonemes 2–5 (*p*s < .001).

The correlation coefficient between the human looking preferences for onset competitors (panel d) and the simulated measure (panel e) was *r*(36) = .33, *p* = .048, suggesting a reasonable fit to the data. Correlation coefficients that focused on the effects of individual factors suggested that ListenIN captured differences in looking preferences between the levels of noise intensity, *r*(24) = .58, *p* = .003, and differences between the levels of nativeness, *r*(18) = .61, *p* < .01, on the looking preferences for onset competitors.

Correlation coefficients for the Input‐based baseline model suggested that it did not capture the looking preferences to onset competitors overall, *r*(36) = .09 *p* = .600. Correlation coefficients focusing on the differences between the levels of individual factors suggested that the baseline model could account for these differences for noise intensity, *r*(24) = .55, *p* = .001, but not for nativeness, *r*(18) = .29, *p* = .24.

### 4.4 Conclusions

Simulation B suggested that ListenIN can account for human performance in the visual‐world paradigm, capturing the effects of noise on human looking preferences in the target and the onset‐phonological‐competitor condition of the task, including differences between the native and non‐native listeners. We conclude that ListenIN can be extended to online task settings.

## 5 Mechanistic analysis of ListenIN's spoken‐word recognition performance

Simulation A demonstrated that ListenIN, a neurocomputational model, was successful at simulating the effects of noise on spoken‐word recognition in an offline task. Simulation B showed that using the same stimuli and the same parameter settings as simulation A, the model reproduced data patterns for the effects of noise on human participants’ eye‐movements in an online visual‐world task. The successful simulation of both data patterns was a prerequisite for investigating the emergence of these effects in the computational model. Therefore, in the next step, we examined the internal workings of the model as it performed an online spoken‐word recognition task (in Dutch) in clean listening conditions and in the presence of noise. With this analysis, we aimed to study the effects of noise on the number of competitors involved in phonological competition and the viability of accounts positing “many‐additional‐competitors” or “single‐strong‐competitor” (Chan & Vitevitch, 2015) scenarios.

We refer to this analysis as mechanistic, in the sense that it uncovers processing mechanisms by which the model recognizes spoken words that are presented incrementally in the input layer, and shows how these mechanisms are affected by noise to yield performance similar to human native and non‐native listeners. The mechanistic analysis is based on a set of online model‐based measures (“neurocomputational proxies”), which, unlike the looking preferences in simulation B, refer to the full training set of the model (not just the four stimuli of the visual‐world paradigm). Furthermore, the mechanistic analysis is not restricted to the output layer of ListenIN, which provided the model‐based measures of spoken‐word recognition in simulations A and B. Here, we also examine activation states across the different hidden layers of the neural network (Fig. 1). This is to shed light on the processing and the representation of incrementally presented input activation patterns in ListenIN, and investigate how the added noise to input phonological representations cascades across the network resulting in the effects observed in simulations A and B. The measures for the mechanistic analysis are computed for both the native and non‐native versions of ListenIN, that is, similar to simulations A and B.

Finally, the mechanistic analysis is also applied to explore the effects of noise on phonological competition interlinguistically, that is, to examine competitors from the nontarget language. This type of competition has not been addressed in the visual‐world paradigm used in simulation B, thus, ListenIN offers an extension of Scharenborg et al. (2018).

### 5.1 Noise leads to lower accuracy during online spoken‐word recognition

How did the presence of background noise affect the ability of ListenIN to recognize words during their incremental presentation?

**Fig 6:**
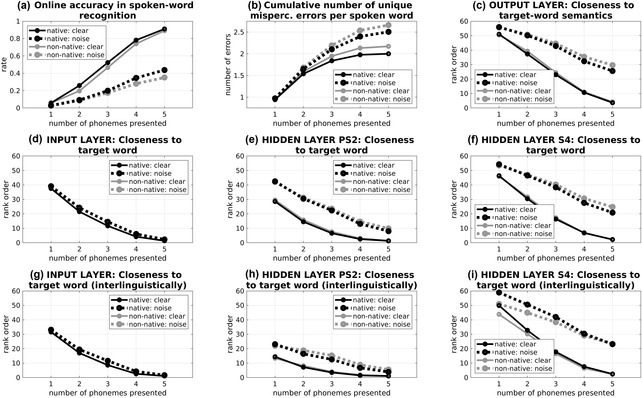
Analysis of ListenIN's performance during online spoken‐word recognition. Panel a shows online accuracy in spoken‐word recognition; panel b shows the cumulative number of unique erroneous responses per spoken word; and panel c shows the rank order of similarity to target‐word output semantics activation. Panels d–f refer to activation states in the input, the composite hidden (PS2), and the semantics hidden S4 layer, correspondingly, and show the rank order for similarity to target‐word internal activation patterns of Dutch words. Panels i–g show variants of the rank‐order measures in panels d–f (correspondingly) referring to interlinguistic competition. Black ink corresponds to the native Dutch ListenIN and gray ink to the non‐native ListenIN. Continuous lines correspond to the clean listening condition and dotted lines to the presence of random noise (*SD* = 0.75).

To address this question, we examined *online accuracy in spoken‐word recognition* (Fig. 6a), which is an online version of accuracy in Scharenborg et al. (2018) and simulation A. Online accuracy was computed as the percentage of stimuli words for which the output semantics of ListenIN (after the partial presentation of this word up to a given timestep) were closer in the Euclidean space to the semantics of the target word than to the semantics of any other Dutch word from the model's vocabulary.

As shown in Fig. 6a, the presence of background noise resulted in detriments in *online accuracy in spoken‐word recognition*. In particular, in native ListenIN and in the absence of noise (thin black continuous lines), this measure was at near‐zero levels (*M* = 0.06) after the presentation of the first phoneme (timestep 1) and approximated ceiling performance (*M* = 0.91) at timestep 5. In the presence of random noise (thick black dotted lines, *SD* = 0.75), accuracy in spoken‐word recognition in native ListenIN was overall lower than in the clean listening condition, reaching 0.44 at timestep 5. In the non‐native version of ListenIN (gray lines), online accuracy was, uniformly, slightly lower than the native version in both clean listening conditions and in the presence of noise.

### 5.2 Noise results in more unique misperceptions during online spoken‐word recognition

How did the presence of background noise affect misperception errors in online task settings?

We addressed this question by measuring the *cumulative number of unique misperception errors* (Fig. 6b), defined as the number of spurious competitors that have been engaged in the phonological competition up to a given timestep. Effectively, this measure is an online version of the unique erroneous responses measure in Scharenborg et al. (2018) and simulation A, which captures response variability across the presentation of spoken words.

The *cumulative number of unique misperception errors* was computed using Euclidean distances between output semantics activation in ListenIN and all individual semantics patterns in the training set. For example, if ListenIN was presented with the target word *dollar* (dollar) and the test stimuli that better matched the output semantics activation was *deur* (door) at timestep 1, *dolfijn* (dolphin) at timesteps 2 and 3, and *dollar* at timesteps 4 and 5, then the cumulative number of unique erroneous responses for the target word was 1 at timestep 1, and 2 at all subsequent timesteps (no increases applied after a word was recognized successfully).

As shown in Fig. 6b, the presence of background noise resulted in increases in the *cumulative number of unique misperception errors*. More specifically, in the native version of ListenIN, the cumulative number of unique misperception errors per spoken word (panel b) in the absence of noise (thin black continuous lines) started at a value of 0.97 at timestep 1 and increased to 2.00 at timestep 5. The increasing trend suggested that multiple nontarget competitor words (2.00 on average) were engaged in lexical competition at different timesteps of spoken‐word recognition. The cumulative number of unique misperception errors per spoken word was higher in the presence of noise in native ListenIN (thick black dotted lines), with an average value of 2.51 at timestep 5. A similar increase was also observed in the non‐native ListenIN (gray ink), in which the cumulative number of unique erroneous responses was uniformly higher compared to the native version.

### 5.3 Output layer: More competitor words outperform the target word in lexical competition in the presence of noise

How strong of a competitor was the target word during phonological competition and how did noise affect its strength?

We addressed this question by examining the *closeness of output activation to target‐word output semantics* (Fig. 6c). This measure is an extension of online accuracy. In particular, while online accuracy in spoken‐word recognition refers to whether or not the model's output semantics was closer to the target‐word semantics (binary outcome), the closeness measure is a rank order, which refers to the number of spurious words that matched ListenIN's output semantics better than the target word.

The *closeness (of output activation) to target‐word output semantics* was calculated using Euclidean distances between output semantics at a given timestep and all individual semantics patterns in ListenIN's training set. The distances were sorted in ascending order, and if the rank order of the distance between ListenIN's output semantics and the target‐word semantics was equal to 1, ListenIN had recognized the target word accurately. However, if the rank order of this distance was greater than 1 (misperception error), this was taken to suggest that *rank order − 1* spurious words were engaged in phonological competition and outperformed the target word at this time step.

As shown in Fig. 6c, the *closeness to target‐word output semantics* in the native version of ListenIN in clean listening conditions (thin black continuous lines) was on average 51.03 in timestep 1 and decreased to 3.62 at timestep 5. Thus, the target word was a relatively weak competitor, which at the onset of spoken‐word‐recognition, was preceded by 50.03 words on average in this measure of competitive strength; but a stronger competitor and most often a winner toward the end of spoken‐word recognition (mean rank order approaching 1.00 at timestep 5).

In the presence of background noise, in native ListenIN (thick black dotted lines), the *closeness to target‐word output semantics* was higher than in the clean listening conditions, suggesting that more spurious words were stronger than the target word in phonological competition. The difference in rank order between clean and noisy listening conditions was more pronounced at later timesteps of online‐spoken word recognition (mean rank order in timestep 5 was 25.58 in noise). A similar increase in this rank order of the similarity between target‐word semantics and output semantics due to noise was also observed in the non‐native version of ListenIN (gray ink), in which this measure was marginally higher.

### 5.4 Input layer: Noise increases the number of competitors engaged in phonological competition

How did the effects of background noise on phonological competition manifest at the input layer?

We examined the effects of noise on processing dynamics at the input layer by extending the rank‐order approach to incrementally presented input phonological representations. In particular, we developed a rank‐order measure for the input layer, referred to as *closeness to target‐word input phonology* (Fig. 6d and e). This measure taps on phonological competition in terms of the similarity between input phonological activation with ListenIN's phonological representations (i.e., similar to the Input‐based model used in simulations A and B).

The *closeness to target‐word input phonology* was computed measuring Euclidean distances between partially presented phonological patterns (e.g., “*d—–*,” corresponding to “*dollar*” (dollar) at timestep 1) and fully presented Dutch phonological forms (e.g., target: “*dollar*,” nontarget: “*dolfin*”). If the Euclidean distances suggested that the top three matches were “*deur*” *(door)*, “*dollar*” (dollar), and *dolfijn* (dolphin), respectively, then the rank‐order measure for closeness to input phonology was 2.

With regard to the clean listening condition, panel d in Fig. 6 shows that the rank‐order for the closeness to *target‐word input phonology* measure decreased progressively from 37.59 at timestep 1 to an average of 1.37 words at timestep 5. The presence of noise resulted in a marginal increase in the *closeness to target‐word input phonology* rank order (dashed line marginally above the continuous line in Fig. 6d). This was a counterintuitive result. The addition of random noise degraded the input vector; however, in terms of closeness to full input patterns, the number of Dutch words that outperformed the input pattern in phonological competition was highly similar to the clean condition. The rank‐order values in the non‐native version overlapped with the native version in plot d.

### 5.5 Hidden layers PS2 and S4: More competitor words outperform the target word in lexical competition in noise

How did the effects of background noise on phonological competition manifest in the hidden layers of the neural network?

To address this question, we extended the rank‐order approach to activation states developed in the composite bottleneck hidden layer PS2 (“bottleneck”) and the hidden semantics layer S4 (Fig. 1). These internal activation states offer insights into abstracted representations of linguistic knowledge that the model learns and uses to produce output semantics patterns. These hidden layer representations are abstracted in the sense that, internally, the model does not encode all the information presented on the input layer, but focuses on features that the network has discovered to be relevant to the tasks on which it has been trained.

More specifically, we developed measures of *closeness to target‐word internal activation states*. These were computed similarly to their input‐layer counterparts (Section 5.4). For a given layer (“bottleneck” hidden layer PS2 or hidden semantics layer S4), we estimated a rank order based on the closeness of internal activation states developed in ListenIN during the incremental presentation of a word to internal activation states elicited by the (full) presentation of phonological patterns corresponding to the target word and all other words, presented together with input semantics. The rank‐order measure was taken to be a proxy for the extent to which the target word was “activated” (in comparison to nontarget words) in ListenIN at a given timestep and within a given layer. For example, if the rank order of the target word was 15, it was taken that 14 spurious words were more “activated” than the target word.

Panels e and f in Fig. 6 show rank‐order measures for the competition between the target word with other Dutch words (intralinguistically) within layers PS2 and S4. In the presence of background noise (dotted lines), the number of Dutch words that were closer to internal activation states of ListenIN (and hence more “active”) compared to the target word was higher than the clean condition. The number of words considered for recognition was also marginally higher in the non‐native (gray lines) than in the native model (black lines).

In hidden layer PS2 (panel e), the effects of noise were more pronounced in timestep 1 than timestep 5, that is, when phonological forms were presented fully. However, in the hidden semantics layer S4 (panel f), the effects of noise increased toward the final timesteps. Thus, even though the network was efficient in processing noisy fully presented phonological forms in the composite hidden layer (reduction of the effect of noise in timestep 5 in Fig. 6e), in the higher hidden semantics layer S4, which feeds to the output semantic layer, the detrimental effects of noise persisted throughout the presentation of spoken words.

### 5.6 Noise increases phonological competition interlinguistically

Was there any competition from words from the nontarget language and how was this affected by noise?

We studied interlingual competition using variants of the closeness measures for the input and the hidden layers (Sections 5.4 and 5.5) that referred to English words (nontarget vocabulary). These are presented in panels g–i of Fig. 6. Competition from English was overall lower than competition from Dutch; however, this difference decreased in higher levels of the neural network, that is, layers involved in computations supporting the mapping of phonological forms to semantics, which are shared between the English and the Dutch vocabularies. Furthermore, in the hidden semantics layer S4 (panel i), interlingual competition (from English) was lower in the non‐native version of the model (gray lines) than the native LestinIN, an effect that likely reflected the relatively high exposure of the non‐native model to the English vocabulary (representations of English words were learnt better and were thus less confusable with noisified Dutch words).

With regard to the effects of noise on intralingual competition, these were similar to intralingual competition as suggested by the consistent pattern of increases in the measures shown in panels d–f and g–i, correspondingly.

### 5.7 Effects of noise on the processing of individual words

What are the effects of noise on the way ListenIN processes individual words?

To address this question, we conducted a representational similarity analysis (RSA; Kriegeskorte, Mur, & Bandettini, 2006; Mehrer, Spoerer, Kriegeskorte, & Kietzmann, 2020; see also Devereux, Clarke, Marouchos, & Tyler, 2013; Kell, Yamins, Shook, Norman‐Haignere, & McDermott, 2018; Magnuson et al., 2020) on activation states across the ListenIN neural network during Dutch spoken‐word recognition. An RSA quantifies the extent to which two different processing systems (in input, output, size, modality, etc.) respond similarly to the same set of stimuli. A high similarity suggests that computations in the two systems reflect (represent or are sensitive to) the same between‐stimuli similarity structure (Magnuson et al., 2020).

Here, we employed RSA to quantify the extent to which activation states developed in different layers of ListenIN reflected the similarity structure embedded in the phonological and semantics representations of ListenIN's training set (see Section 2.2). More specifically, we measured the extent to which the different layers reflected the training set's phonological and semantics structure during the incremental presentation of Dutch phonological forms (i.e., timesteps 1–5), and examined how this relationship was affected by the addition of random noise to the phonological input. Furthermore, similar to simulations A and B, we considered both the native and non‐native versions of ListenIN.

**Fig 7:**
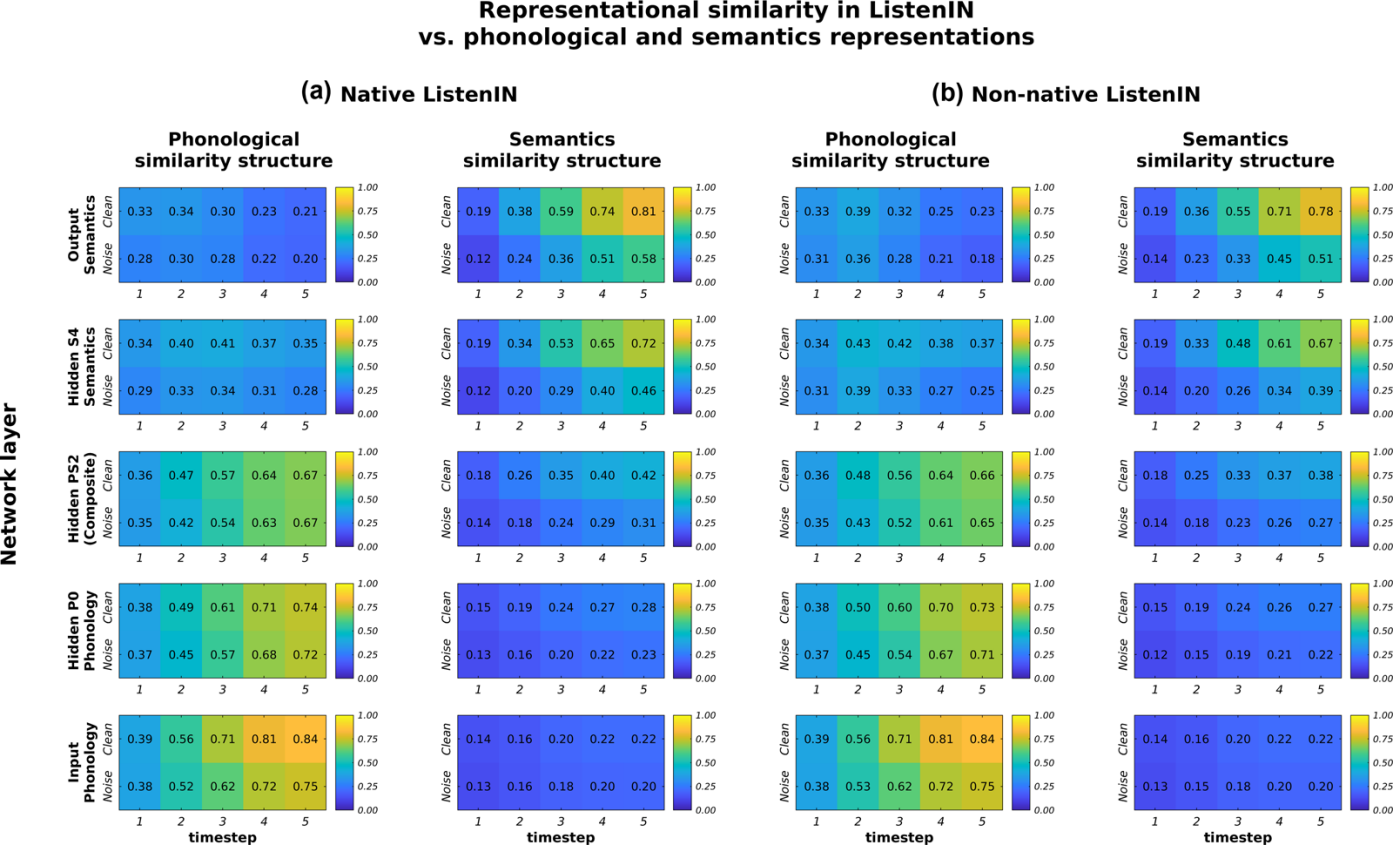
Relationship between representational similarity in ListenIN's and the similarity structure in the phonological and semantics representations. The colored squares show the strength of the correlation coefficients between the (representational dissimilarity matrices) RDMs for different layers in ListenIN and the RDMs for the training set's phonological and semantic representations. Each panel shows correlation coefficients for timesteps 1–5 of the incremental presentation of input phonology and for clean and noisy (*SD* = 0.75) listening conditions (top and bottom row in each panel). Warmer colors suggest higher similarity and hence that activation states in ListenIN are consistent with regularities embedded in the model's representations (phonological or semantics) to a greater extent. (a) Native ListenIN; (b) non‐native ListenIN. The two columns of panels in subplots a and b show comparisons with phonological and semantics RDMs, correspondingly. Rows correspond to different layers of the neural network architecture (starting from the bottom: input, hidden layer P0, hidden layer PS2 [composite], hidden layer S4, and output layer).

The RSA was carried out as follows. First, we generated word‐to‐word (121×121) representational dissimilarity matrices (RDMs) for each layer and for each timestep of the incremental presentation of Dutch phonological forms. For a given layer and timestep, an RDM column included cosine similarity distances between activation patterns resulting from the incremental presentation of the target word and activation patterns corresponding to the other Dutch words (all words presented up to the timestep in focus). Subsequently, we compared these RDMs referring to ListenIN layers with RDMs computed for the training set's phonological and semantics representations. These comparisons were made using Pearson correlation coefficients. The coefficients are visualized in the colored cells of Fig. 7, with warmer colors suggesting that the RDMs in ListenIN are more similar to RDMs for the training set's phonological and semantics representations.

As shown in the bottom rows of Fig. 7, the input phonology layer and the hidden layer P0 reflected a great deal of the similarity structure embedded in the training set's phonological representations (correlation coefficient = 0.84 at timestep 5 in the clean condition), and less of the similarity embedded in the semantics representations (correlation coefficient = 0.22 at timestep 5 in the clean condition). The opposite holds for the higher hidden layer S0 and the output layer (top rows in Fig. 7), in which ListenIN's activation states were more sensitive to the similarity structure within semantics rather than the phonological representations (e.g., output semantics at timestep 5: correlation coefficient with phonology = 0.21, correlation coefficient with semantics = 0.81). In the intermediate hidden layers P0, PS2, and S4, there was a gradient pattern of correlation coefficient values between the input and output layers.

Irrespective of whether a given layer is more sensitive to the similarity structure of phonological or semantics representations, the correlation coefficients between RDMs for ListenIN's layers and RDMs for the phonological and semantics representations increased between timesteps 1 and 5. This suggests that as incremental spoken‐word presentation progressed, ListenIN's activation states became increasingly sensitive to the similarity structure of the training set.

The effects of noise on the similarity of RDMs for ListenIN's layers and RDMs for the training set's phonological and semantics representations can be inspected by comparing the two rows of the individual subplots of Fig. 7 (top: clean condition; bottom: noise). With regard to the phonological representations (panels in the first main column of Fig. 7), in the input phonology layer (lower panel), the addition of noise resulted in lower correlation coefficients (reducing from 0.84, in the clean condition, to 0.75 in noise, at timestep 5). This effect was not present in the hidden phonology P0 and the composite hidden layer hidden PS2, as well as the hidden S4 and the output semantics.

Thus, hidden layers in ListenIN processed noisified incrementally presented input patterns very similarly to incrementally presented input patterns without noise (in terms of correspondence with the phonological similarity structure embedded in the training set), as if they were filtering out some of the degradation of the phonological input due to noise.

With regard to the effects of noise on the similarity between RDMs for ListenIN's layers and the RDM for the training set's semantics representations (panels in the second column in Fig. 7), in the input layer, the correlation coefficient decreased marginally due to the addition of noise (clean: 0.22; noise: 0.20). However, this effect became increasingly stronger across the higher layers of the network (hidden phonology P0, hidden PS2, hidden semantics S4, and output layer), which were also more sensitive to the training set's semantics similarity structure. In the output layer, noise resulted in considerably lower correlation coefficient values (e.g., timestep 5: correlation coefficient = 0.58 vs. 0.71 in the clean condition).

Finally, with regard to the comparisons of RDMs for ListenIN's layers and the RDM for the training set's representations in the native and the non‐native versions of ListenIN (Fig. 7a vs. b), there were relatively small differences between the two versions of ListenIN on the correlation coefficients referring to the phonological similarity structure, but higher differences in correlation coefficients referring to the semantics similarity structure. Otherwise, noise affected the correlation coefficients between the RDMs in ListenIN's layers and the RDMs of the training set representation similarly in the native and the non‐native version.

### 5.8 Summary

In sum, the mechanistic analysis of ListenIN's online spoken‐word recognition performance demonstrated that worse spoken‐word recognition performance in noise was associated with a tendency to produce more variable misperception errors during word presentation. When target words were presented in noise, a larger number of spurious competitor words were engaged in the phonological competition across all timesteps of the incremental presentation of input phonological forms, compared to the clean listening condition. These effects were observed in both the native and non‐native versions of the computational model.

The internal activation states developed in ListenIN during the incremental presentation of noisified phonological forms were more often similar to nontarget Dutch words than to the target word (presented in full). Thus, we observed enhanced phonological competition, reflected in a higher number of nontarget words being more “activated” than the target word during spoken‐word recognition. Noise also reduced the sensitivity of ListenIN to the similarity structure embedded in the training set's phonological and semantics representations.

In the input phonology layer in particular, the presence of background noise affected different model‐based measures of phonological competition in different ways. On the one hand, noise had only a marginal influence on the number of nontarget words that matched incrementally presented input (*Closeness to target‐word input activation*, Fig. 6d). On the other hand, noise had a substantial effect on the correlation coefficients with the phonological similarity structure (RSA, Fig. 7, bottom row). This pattern of results suggests that in the presence of noise, a slightly larger number of spurious competitors––though a quite dissimilar set of phonological forms––were engaged in phonological competition compared to clean listening conditions. Nevertheless, increases in the size of the competitor space due to noise were more pronounced in higher levels of the processing hierarchy, which were involved in the processing of abstracted phonological and semantics information.

During spoken‐word recognition, there was also considerable interlingual competition, from words of the nontarget language. Interlingual competition increased progressively within the neural network's processing hierarchy of spoken‐word recognition and as semantics (a common element of the two vocabularies) became increasingly relevant to the processing carried out in individual layers. The effects of noise on interlingual competition were similar to intraligual competition.

The effects of noise on the neurocomputational proxies were also similar in the native and the non‐native version of ListenIN, suggesting that its account for the effects of noise on phonological competition in ListenIN was not differentiated when exposure to training material varied.

In conclusion, the mechanistic analysis of ListenIN's spoken‐word recognition performance demonstrates, within the neurocomputational modeling framework, that a higher number of spurious words were involved in lexical competition during spoken‐word recognition in the presence of noise. It also showed how results from offline spoken‐word identification (Scharenborg et al. (2018) and simulation A) emerged during the timecourse of spoken‐word recognition in online task settings rather than at decision time.

## 6 Discussion

Computational modeling allows for the explicit examination of the internal states of the comprehension system while it carries out a word recognition task––something that is more challenging to achieve behaviorally. In this study, we used computational modeling to study the effects of noise on the size of the competitor space during the unfolding of the spoken‐word recognition process. We aimed to investigate whether these effects could account for human performance in both offline and online task settings and in listeners with different language proficiency.

Our results on the *rank order of the closeness of the output semantics* (Fig. 6c) showed that in the presence of noise, a larger number of competitor words were engaged in phonological competition throughout the spoken‐word recognition process in ListenIN. This is in line with the “many‐additional‐competitors scenario” (Chan & Vitevitch, 2015), as also observed by Scharenborg et al. (2018), and Poretta and Kyröläinen (2019) for foreign‐accented speech. Furthermore, the involvement of additional spurious words in phonological competition in the presence of noise co‐occurred with higher rates of misperception errors and detriments in spoken‐word recognition accuracy throughout the spoken‐word recognition process. The results of Scharenborg et al. (2018) were obtained during an offline experiment, that is, word identification was only measured after the entire word recognition process, and the misperceptions were considered as a proxy of the number of activated words. The simulation results presented here suggest that the activation of spurious word candidates occurs, indeed, throughout the multiple activation process and not only during recognition (decision). Moreover, the engagement of additional competition in the presence of noise (and the high rates of misperception errors) co‐occurs with––and could thus account for––the prolonged looking preferences for phonological competitors in visual world paradigms (see Poretta & Kyröläinen, 2019, for a similar argument).

Another way in which our findings supported the viability of a “many‐additional‐competitors scenario” was through the internal activation states developed in ListenIN's hidden layers during the incremental presentation of target words. These were often more similar to internal activation states that would have been induced by nontarget rather than target words and this mismatch was taken as a neurocomputational analogue of spurious word representations being “activated” and competing for recognition during spoken‐word recognition. Furthermore, the rank order of the match between a current internal activation state in ListenIN and representations of fully presented words was used to quantify the level of “activation” of target and nontarget words during online spoken‐word recognition. Crucially, a larger number of nontarget words were “activated” and engaged in phonological competition in the presence of noise.

Additionally, the RSA (Fig. 7) suggested that the addition of random noise to input phonological representations resulted in internal activation states that were less sensitive to the phonological and semantics regularities in the model's training set compared to the clean listening condition. This appeared to be a mechanism by which ListenIN generated higher levels of nontarget‐word activation and lexical competition in the presence of background noise. The locus of this mechanism was in higher levels of the hierarchy of the neural network: Worse spoken‐word recognition performance in the presence of noise did not arise from the degraded speech input per se, but from its mapping onto semantics representations.

With regard to the effects of noise on native and non‐native listening, our results suggest that these are similar in native and non‐native listeners. Scharenborg et al. (2018) have suggested that similar effects arise because the underlying native and non‐native spoken‐word recognition systems are fundamentally the same. Our results show the viability of this proposal. ListenIN simulated native and non‐native data based on the same cognitive architecture, which was differentiated only through exposure (see also Scharenborg & van Os, 2019; Scharenborg et al., 2018). This approach is in line with accounts suggesting that worse spoken‐word recognition performance in non‐native compared to native ListenIN reflects imperfect language knowledge (cf. García Lecumberri et al., 2010) and limitations in language‐dependent processing (e.g., difficulties in extracting linguistic information; Krizman, Bradlow, Lam, & Kraus, 2017).

ListenIN introduces three main novelties in computational modeling work carried out in the field of spoken‐word recognition. First, it is the first model to address the effects of background noise on phonological competition and spoken‐word recognition. Second, it is the first model to address the effects of noise in both native and non‐native listeners. Third, it is novel in exploring the temporal dynamics of the effects of noise through the analyses of internal activation states.

ListenIN exhibited good potential for generalizability as it captured human performance in an offline and an online task carried out in different modalities (offline: auditory; online: auditory and visual). The model did so using the same stimuli and networks, without any further parameter tuning. Furthermore, ListenIN is inclusive of native and non‐native spoken word recognition. Finally, as ListenIN incorporates a learning process, it can be extended to (bi/multilingual) lexical development.

The model yielded two counterintuitive predictions, which could be tested in future studies with human listeners. The first prediction refers to the pattern shown in Fig. 6d, according to which the presence of background noise did not drastically change the closeness of incrementally presented phonological forms to the corresponding full phonological pattern (rank order in relation to other full phonological patterns). This effect manifests itself as continuous and dotted lines overlap in Fig. 6d (note, however, the input‐layer's correlation coefficients with the phonological similarity structure were lower in the presence of noise, Fig. 7). This result suggests that worse spoken‐word recognition performance in the presence of background noise could emerge even if the speech signal is similar to the same number of words (but not necessarily the same words) in clean listening conditions and noise. Artificial‐language‐learning paradigms and speech analysis methods can be used to test this prediction.

The second prediction refers to the pattern shown in panels g–i in Fig. 6, which refer to interlinguistic competition. This can be weaker than intralinguistic competition in the early phases of spoken‐word recognition, which are primarily driven by acoustic properties of the speech signal. However, interlinguistic lexical competition is strengthened in later phases, which involve the processing of semantic information. This prediction could be tested with interlingual visual‐world paradigms.

This modeling study is not without shortcomings. In terms of fitting the human data, ListenIN overestimated the human looking preferences to targets (twice as high compared to the model). It is likely that this limitation is related to the limited vocabulary of 242 words of ListenIN, which makes the recognition of target words in a visual‐world‐paradigm setting easier than in humans. Other modeling (over)simplifications are also relevant to achieving a better fit to human data. First, the feature and slot‐based representations of phonology and the implementation of speech‐shaped noise as a random vector added to the binary phonological patterns overlook key characteristics of speech. Future versions of ListenIN should employ representational schemes closer to speech signals (Norris & McQueen, 2008) or, preferably, actual speech signals (e.g., Kell et al., 2018; Magnuson et al., 2020; Nenadic, Bosch, & Tucker, 2017; Scharenborg, 2010). Similarly, implementations of noise should be consistent with known effects of noise on the acoustic characteristics of the speech input or speech signals superimposed with background noise (Kell et al., 2018).

Another direction for future modeling work is using architectures with recurrency, as these are better‐suited to address the incremental nature of speech input and have supported important advances in automatic speech recognition (Yu & Deng, 2015). For example, long short‐term memory nodes, which can support computational models that map speech input to semantics and capture the temporal dynamics of phonological competition (EARSHOT: Magnuson et al., 2020; see also Duta & Plunkett, 2021), could be employed to address the effects of noise.

With regard to variations in language proficiency, ListenIN was limited to a single parameter for the rate of exposure to the English and the Dutch vocabulary during training, while it was also not fully aligned with the human data in terms of the nontarget language in non‐native ListenIN in simulation B (ListenIN: English/humans: German). However, native and non‐native language learning involves considerable individual variability, in the mode, timing, and exposure to the two languages, or in language typology (Mattys et al., 2012). All this variability needs to be incorporated in future extensions of ListenIN.

## 7 Conclusion

ListenIN offered insights into the temporal dynamics of phonological competition in spoken‐word recognition in the presence of background noise based on the neural‐network modeling framework. The two computational simulations and the mechanistic analysis of the model demonstrated the viability of the proposal that additional spurious candidate words are involved in lexical competition in the presence of noise, resulting in delays in the resolution of competition and spoken‐word recognition, and in more variable misperceptions of noisy speech signals.

## Supporting information


**Fig. S1**. Results from control simulation A, where native listeners are modeled with ListenIN trained on a monolingual English vocabulary.
**Supplementary Material S2**. Control simulation B. Target empirical data (panels a and e) and modeling results from ListenIN (panels b and d) and the Input‐based model (panels c and f) in online spoken‐word identification.
