# Supplementary material for: The Presence of Background Noise Extends the Competitor Space in Native and Non‐Native Spoken‐Word Recognition: Insights from Computational Modeling
Source: Cogn Sci. 2022 Feb 21;46(2):e13110. doi: 10.1111/cogs.13110 (PMC9286693; doi:10.1111/cogs.13110)
Supplement: Supplementary file 1 — Fig. S1. Results from control simulation A, where native listeners are modeled with ListenIN trained on a monolingual English vocabulary. Supplementary Material S2. Control simulation B. Target empirical data (panels a and e) and modeling results from ListenIN (panels b and d) and the Input‐based model (panels c and f) in online spoken‐word identification. [file COGS-46-0-s001.docx]

**SUPPLEMENTARY MATERIAL**

**The presence of background noise extends the competitor space in native and non-native spoken-word recognition: insights from computational modeling.**

## S1. Parameters of the autoencoder architecture

The parameters of the autoencoder network in the current version of ListenIN were as follows. The input and the output layers consist of 292 units representing phonology and 300 units representing semantics, respectively. There are five hidden layers. The first hidden layer is bipartite and consists of two banks of 250 units, one fully connected to input phonology and another fully connected to input semantics. The second hidden layer (400 units) is composite and fully connected to the first layer. The third hidden layer comprises 150 units. The autoencoder is symmetric, therefore the fourth hidden layer is identical to the second hidden layer, and the fifth hidden layer is identical to the first hidden layer.

## S2. Phonological scheme

This scheme encoded 51 distinct phones, 20 vowels and 31 consonants, corresponding to the combined phonemic inventories of Dutch and English. These phones were encoded with 22 binary phonological features: consonant, vowel, obstruent, sonorant, aspirated, voiced, plosive, continuant, nasal, lateral, rhotic, strident, labial, coronal, dorsal, glottal, distributed, high, mid, low, retracted, and long (Clark, Yallop & Fletcher, 2006). As the phonological features were binary, an ‘underspecified’ value, for example, to model that the LONG feature is phonologically contrastive in Dutch but not in English, was not considered (zero values were used as a default). For the implementation of the phonological scheme see “Training Set.xlsx” in Supplementary materials.

## S3. Training the neural network

## Weight initialization with pre-training

The weights of the deep autoencoder were initialised using the method of Hinton and Salakhutdinov (2006). Weights between the individual layers of the deep autoencoder (e.g., between Input Phonology and Hidden P0) were trained separately, within shallow (one hidden layer) autoencoders (for a demonstration of this method see Hinton & Salakhutdinov, 2006). Pre-training was implemented in MatLab using the neural network toolbox (The MathWorks Inc., 2012), using the Scaled Conjugate Gradient Algorithm (Møller, 1993) with sparsity regularization (Olshausen & Field, 1997) and the following parameters: 2000 epochs, L2 WeightRegularization = 0.01, SparsityProportion = 0.10.

## Weight fine-tuning with interleaved training phases

After initialization, the weights of the deep network were fine-tuned. Weight fine-tuning lasted for 1000 epochs and used the backpropagation algorithm (Rumelhart, Hinton, & Williams, 1986) with the cross-entropy error criterion (Hinton, 1989; learning rate, lr = 0.05, zero momentum). The following three phases were used: (A) the phonology-to-phonology phase, in which only input phonology was presented to the network, which was trained to produce the same phonological pattern in the output layer (no changes applied to the semantics pathway during this phase); (B) the semantics-to-semantics phase, which focused on learning of the semantics pathway; and (C) the mixed phase, in which both phonology and semantics were presented in the input layer and learning happened across the whole network.

Each epoch had 242 (2*121) training sweeps. In each sweep, a randomly-chosen word was presented to the network. A probabilistic training regime controlled for the ratio of English and Dutch words to which the networks were exposed (Filippi et al., 2014). For any given word, one of the following two sequences of the learning phases (see above) was applied: (A)−(C)−(A) or (B)−(C)−(B) (chosen randomly). To include differences in the frequency of occurrence of individual words, assumed to be similar in English and Dutch, and their impact on learning, the weight changes estimated by the learning algorithm were multiplied by the log-transformed CELEX word frequencies (Baayen, Piepenbrock, & Gulikers, 1995) (as in Plaut, McClelland, Seidenberg, & Patterson, 1996).

## Weight fine-tuning with denoising

A denoising-autoencoder training technique (see Vincent et al., 2008; Zur et al., 2009) was used to prevent overfitting of the neural networks through the artificial expansion of the training data (by presenting multiple variations of training data). Denoising was implemented by injecting random noise probabilistically in half of the training sweeps and to the phonological part of the input or the semantics part or both. In all cases, the network should output denoised activations. The noise that was added to a given input unit’s activation for the purposes of fine-tuning had a zero mean and SD equal to 7 times the SD of the average unit’s activation across the representations of the training set. It should be noted that this type of noise is different from the noise implemented in Simulations A and B (discussed below) to model the effects of background noise on spoken-word recognition. Here the aim was to enable (synergistically with the techniques discussed in S3.1 and S3.2) the neural network to learn mapping of phonological forms onto their semantics.

## S4. Simulation A: Simulating native listeners with a monolingual version of ListenIN

In the simulations reported in the main text of the manuscript, the training set of the native and non-native versions of ListenIN include both the English and the Dutch vocabulary. This implied that the native and the non-native version were aligned in terms of the sheer number of mappings phonological forms and meanings they are exposed to during training. Thus, any differences between the native and non-native conditions were not driven by vocabulary-size differences, but from differences in the quality of vocabulary knowledge emerging from the differential exposure to the two vocabularies.

We have performed control simulations in which non-native listeners were modelled with a monolingual version of ListenIN and exactly the same other parameter settings. Supplementary Fig. 1 shows results from Simulation A. These were highly similar to the results reported in the main text of Simulation A and all the reported effects were the same in the control simulation.

For example, the quantitative comparisons between the modelling results on accuracy (panel b) with the corresponding human data (panel a), the Pearson correlation coefficient was r(16) = 0.96, p < .001, suggesting an excellent fit. Pearson correlation coefficients comparing differences between the levels of noise intensity also suggested an excellent fit to the human data, r(12) = 0.92, p < .001. The same held for correlation coefficients comparing differences between the levels of noise position, r(8) = 0.98, p < .001, and group, r(8) = 0.94, p < .001, on accuracy. For the number of alternative responses, the Pearson correlation coefficient between human data and modelling results on the number of unique misperception errors was *r*(16) = 0.92, *p* < .001, suggesting an excellent fit. The model also captured differences between the levels of noise intensity, *r*(12) = 0.93, *p* < .001, noise position, *r*(8) = 0.95, *p* < .001, and group, *r*(8) = 0.71, *p* = .048.


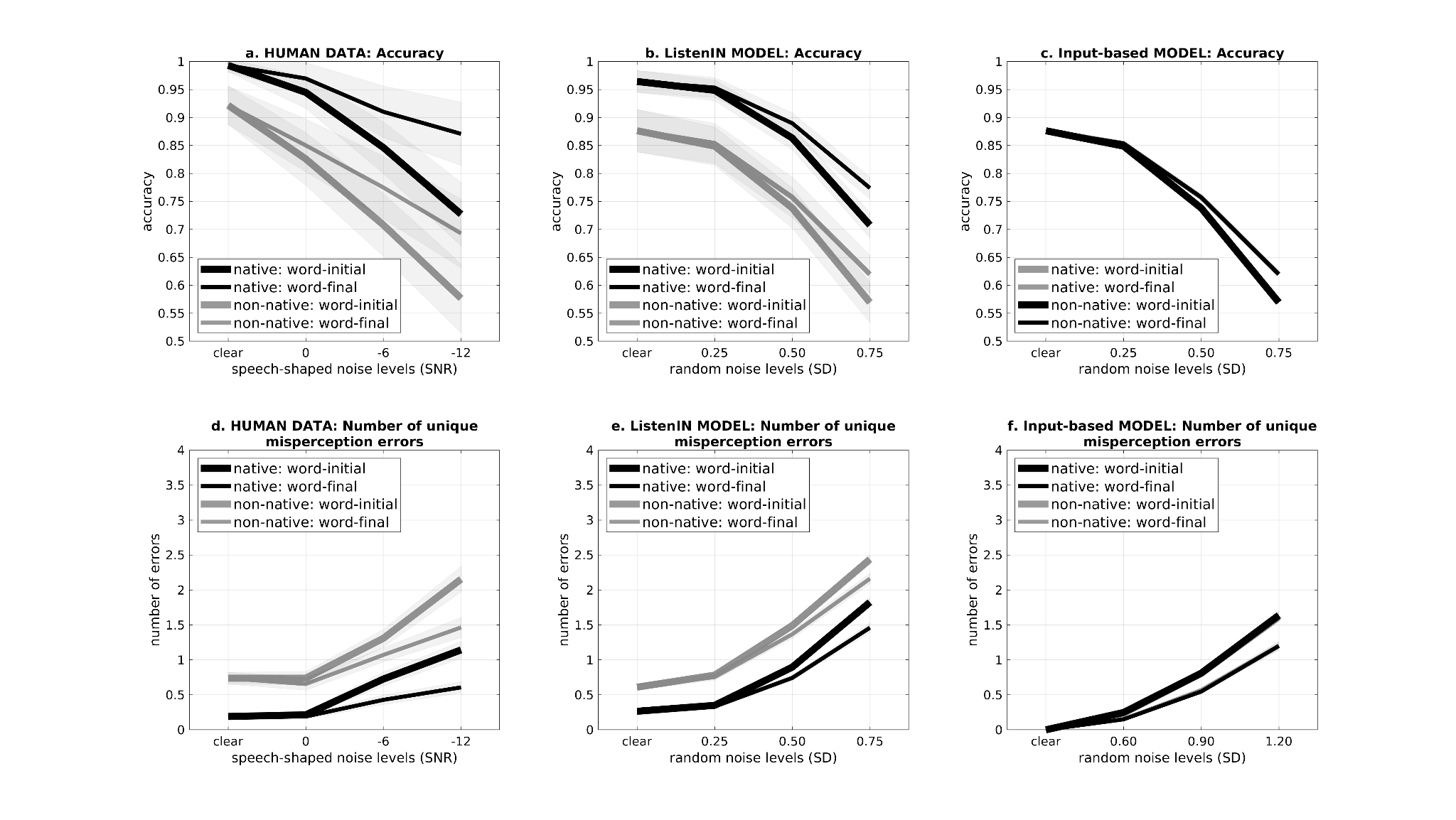


Supplementary Fig. S1. Results from control Simulation A, where native listeners are modelled with ListenIN trained on a monolingual English vocabulary. Target empirical data (panels *a* and *d*) and modelling results from ListenIN and the Input-based model (panels *b, c, e* and *f*) in offline spoken-word identification. The top panels (*a* to *c*) present overall accuracy for different noise intensities; the bottom panels (*d* to *f*) present the number of unique misperception errors for different noise intensities. Noise intensity refers to SNR values for speech-shaped noise in the human data; and to the standard deviation of the added noise in ListenIN and the Input-based model. Black ink shows performance of native listeners; grey ink shows performance of non-native listeners (blank and grey lines overlap in *c* and *e*). Thick lines correspond to the word-initial noise condition; thin lines correspond to the word-final noise condition. Error bars show 1 SEM.

##

## S5. Simulation B: Simulating native listeners with a monolingual version of LIstenIN

We have performed a control simulation B, modelling native Dutch listeners with ListenIN being exposed to the Dutch vocabulary only. Again, the results, shown in Supplementary Fig. 2, were highly similar to the simulation reported in the main text of the manuscript and all reported effects were the same as in Section 4.3.

For example, for the looking preferences for targets, the correlation coefficient between the human data (panel a) and the modelling results (panel b) was *r*(36) = 0.95, *p* < 0.001, suggesting an excellent fit − though, notably, looking preferences to targets in ListenIN are twice as high compared to the model. Correlation coefficients focusing on differences between the levels of individual factors were significant for noise intensity, r(24) = 0.67, *p* < 0.01,and presented a non-significant trend nativeness, r(18) = 0.43, *p* = 0.074.

For onset competitors, the correlation coefficient between human data and simulated measures was *r*(36) = 0.31, *p* = 0.068 (non-significant teend). Again, ListenIN captured differences in looking preferences between the levels of noise intensity, r(24) = 0.53, *p* = 0.008, and differences between the levels of nativeness, r(18) = 0.62, *p* = 0.006, on the looking preferences for onset competitors.


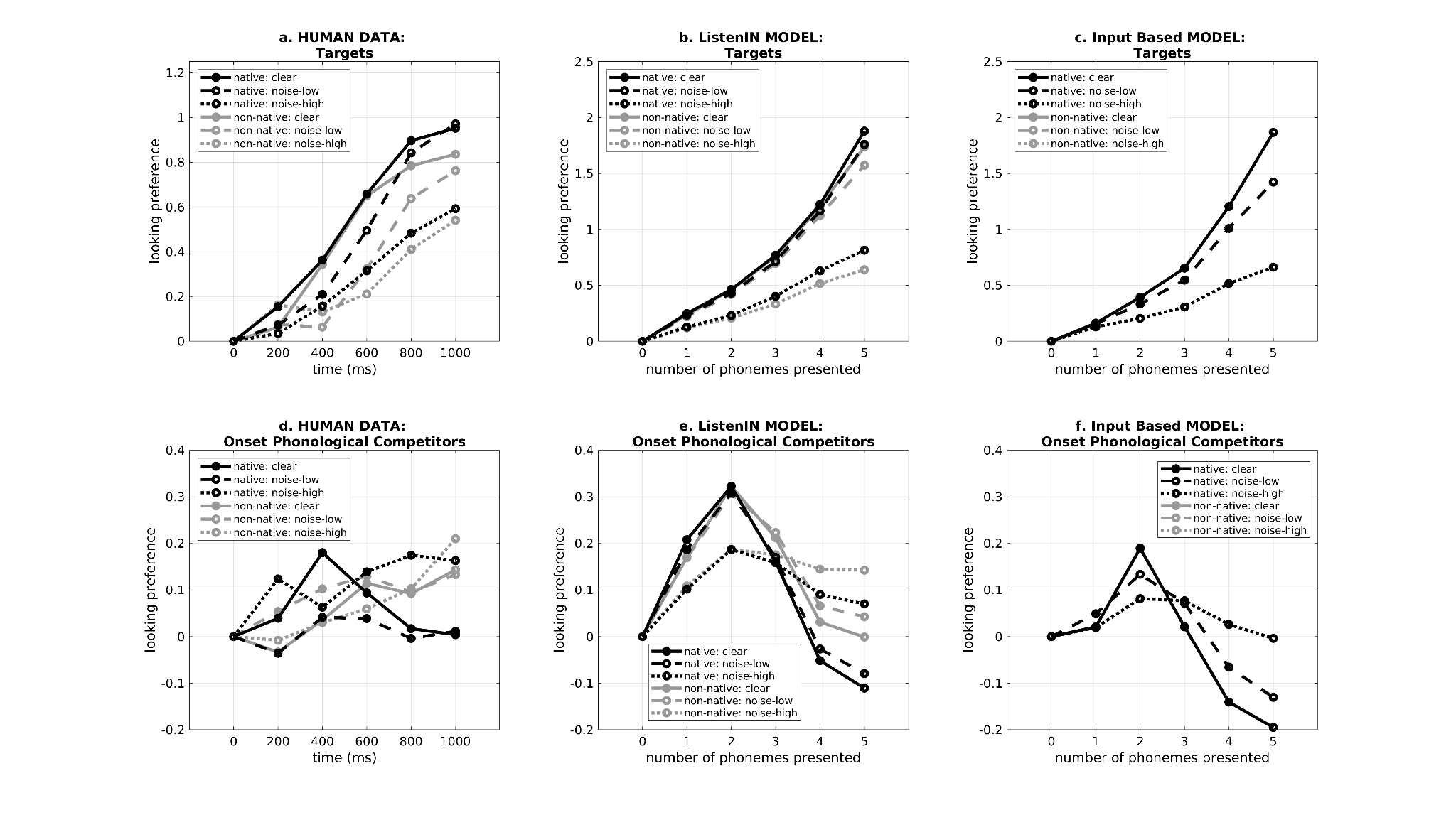


Supplementary Fig. S2. Control Simulation B. Target empirical data (panels *a* and *e*) and modelling results from ListenIN (panels *b* and *d*) and the Input-based model (panels *c* and *f*) in online spoken-word identification. The top panels (*a* to *c*) present looking preferences for target words; the bottom panels (d and f) present looking preferences for onset phonological competitors. Time is measured in ms in the human data and in the number of phonemes that have been incrementally presented in the modelling results. Black ink shows the performance of native listeners; grey ink shows the performance of non-native listeners (black and grey lines overlap in panels *c* and *f*). Continuous lines correspond to the clean listening condition; dashed and dotted lines correspond to the noisy listening condition (SNR of +3dB and −3dB in humans, added random noise with a standard deviation SD = 0.25 and SD = 0.75 in ListenIN, and SD = 0.60 and 1.20 for the Input-based model - settings from Simulation A).

##

## References

Baayen, R. H., Piepenbrock R., & Gulikers, L. (1995*). The CELEX Lexical Database (Release 2)* [CD-ROM]. Philadelphia: Linguistic Data Consortium, University of Pennsylvania.

Clark, J., Yallop C., & Fletcher. J. (2006). *An introduction to phonetics and phonology* (3rd ed.). Oxford, UK: Blackwell.

Filippi, R., Karaminis, T., Thomas, M. S. C. (2014). Language switching in bilingual production: Empirical data and computational modelling. *Bilingualism: Language and Cognition, 17,* 294–315.

Hinton, G. E. & Salakhutdinov, R. P. (2006). Reducing the Dimensionality of Data with Neural Networks. *Science, 313,* 504–507.

Hinton, G.E. (1989) Connectionist learning procedures. *Artificial Intelligence, 40,* 185–234.

Møller, M. F. (1993). A Scaled Conjugate Gradient Algorithm for Fast Supervised Learning. *Neural Networks, 6,* 525–533.

Olshausen B. A., & Field, D. J. (1997). Sparse Coding with an Overcomplete Basis Set: A Strategy Employed by V1. *Vision Research, 37,* 3311–25.

Plaut, D. C., McClelland, J. L., Seidenberg, M. S., & Patterson K. (1996). Understanding normal and impaired word reading: Computational principles in quasi-regular domains. *Psychological Review, 103,* 56–115.

Rumelhart, D. E., Hinton, G. E., & Williams, R. J. (1986*). Learning internal representations by error propagation.* In D. E. Rumelhart, J. L. McClelland, and the PDP Research Group (Eds.), Parallel distributed processing: Explorations in the microstructure of cognition (Vol. 1: Foundations), pp. 318–362. Cambridge, MA: The MIT Press.

The MathWorks (2012). *MATLAB and Neural Network Toolbox, Release 2012b.* Natick, MA: The MathWorks Inc.

Vincent, P., Larochelle, H., Bengio, Y., & Manzagol, P. A. (2008). *Extracting and composing robust features with denoising autoencoders.* In Proceedings of the 25th International Conference on Machine Learning (ICML-08), pp. 1096-1103.

Zur, R. M., Jiang, Y., Pesce, L. L., & Drukker K. (2009). Noise injection for training artificial neural networks: A comparison with weight decay and early stopping. *Medical Physics, 36,* 4810-18.
